# Fluoroquinolone resistant *Escherichia coli* in community: molecular analyses of resistance genes, virulence factors, and antimicrobial susceptibility test

**DOI:** 10.3389/fmicb.2026.1730332

**Published:** 2026-01-27

**Authors:** Zuleica Naomi Tano, João Gabriel Soncini, Vanessa Lumi Koga, Julia Silva Pimenta, Pedro Olimpio Siqueira Castilho, Maria Julia Onça Moreira, Alanis Cassamassimo Cardoso, Larissa Sugiura, Renata Katsuko Takayama Kobayashi, Nilton Lincopan, Wander Rogério Pavanelli, Eliana Carolina Vespero

**Affiliations:** 1 Infectious Diseases, Department of Internal Medicine, State University of Londrina, Londrina, Paraná, Brazil; 2 Laboratory of Clinical Microbiology, Department of Pathology, Clinical and Toxicological Analysis, Health Sciences Center, State University of Londrina, Londrina, Paraná, Brazil; 3 Department of Microbiology, Biological Science Center, State University of Londrina, Londrina, Paraná, Brazil; 4 Department of Microbiology, Institute of Biomedical Sciences, University of São Paulo, Butantã, São Paulo, Brazil; 5 Laboratory of Immunoparasitology of Neglected Diseases and Cancer, State University of Londrina, Londrina, Paraná, Brazil

**Keywords:** antibiotic resistance, epidemiology, *Escherichia coli*, urinary tract infection, virulence

## Abstract

**Introduction:**

Most urinary tract infections in all populations are caused by gram -negative, facultative anaerobic, uropathogenic *E. coli* (UPEC). Multidrug resistance (MDR) to first line antibiotics recommended for treatment of UTI is a global health problem. The misuse of antimicrobial agents for UTI treatment can lead to selection of bacterial MDR, able to transfer mechanism of resistance through mobile genetic elements (MGEs), such as transposons, integrons and conjugative plasmids. This study aims to characterize MLST, virulence and resistance genes to quinolones and β-lactams of UPEC samples isolated from UTI in community.

**Methods:**

*E. coli* strains were isolated from urine samples from June 2016 to June 2018. The identification and bacterial susceptibility were realized by automated VITEK^Ⓡ^2 system. Enterobacterial Repetitive Intergenic Consensus (ERIC-PCR) was performed to assess genetic similarity. The DNA was used to construct a paired-end library, which was sequenced using the NextSeq platform (Illumina). Genome assemblies was performed by the CLC Genomics Workblench version 7.0. Multilocus sequence type (MLST), resistome and virulome were identified using bioinformatics tools available from the Center for Genomic Epidemiology.

**Results:**

This study analyzed 54 *E. coli* isolates resistant to fluoroquinolones and/or producing ESBLs. High resistance rates were observed for ampicillin (100%), nalidixic acid (96.6%), ceftriaxone (83.3%), cefepime (81.5%), ciprofloxacin (81.5%), norfloxacin (81.5%), sulfamethoxazole/trimethoprim (75.9%), and nitrofurantoin (71.9%). Genomic analysis revealed 25 sequence types (STs), with ST131 being most prevalent (22.2%), and 29 serotypes, notably O25:H4 (16.7%). Furthermore, it also showed β-lactam resistance genes including *blaCTX-M* (77.8%), mainly *blaCTX-M-15* (25.9%), and others like *blaTEM-1B* and *blaOXA-1*. Mutations in *gyrA* (S83L, D87N) and *parC* (S80I) were common (98.1%), along with plasmid-mediated resistance (PMQR), such as *aac(6’)-Ib-cr* (13%). Virulence genes commonly linked to UTIs were detected, with *iss* (72.2%) being the most frequent. Most isolates had up to three virulence genes. Plasmids were detected in nearly all isolates, especially *IncF*.

**Discussion:**

This study reveals the widespread circulation of high-risk, multidrug-resistant UPEC clones in community-acquired UTIs, particularly those belonging to the globally disseminated ST131 lineage. The frequent coexistence of resistance determinants, virulence factors, and IncF plasmids highlights the adaptive success of these strains and their potential for sustained dissemination. These findings emphasize the need for continuous genomic surveillance, rational antimicrobial use, and region-specific treatment guidelines to mitigate the impact of MDR UPEC in the community.

## Introduction

Urinary tract infection (UTI) is the second most common bacterial infection in USA, with 150 million people diagnosed per year. The incidence, severity and recurrence of UTI depend on host factors, such as host’s immunity and gender. Due to anatomical features, women are more susceptible to develop an UTI than men and over 20% of these women presents UTI recurrence despite antibiotic treatment (Flores-Mireles et al., 2015). Uropathogenic *Escherichia coli* (UPEC) is the most common etiology, responsible for 80–90% of all cases (Flores-Mireles et al., 2015; Johnson et al., 2005; Tano et al., 2022).

Epidemiological investigations of UPEC isolates have revealed the ST131 *E. coli* strains from different serotypes (O25: H4, O1: H6 O75: H5 and others) are the most common in community-acquired UTI. Generally, ST131 strains are associated with cephalosporins and fluoroquinolones resistance. The acquisition and expression of extend spectrum *β*-lactamases (ESBL), mainly CTX-M, TEM and SHV, is the main β-lactam resistance in these strains. Moreover, mutations in the genes encoding DNA gyrase (*gyrA/gyrB*) and topoisomerase IV (*parC/parE*), as well as the acquisition of genes such as *qnrA/B*, may lead to fluoroquinolone resistance (Coque et al., 2008; Cagnacci et al., 2008). These acquired resistance mechanisms are mainly mediated through plasmids which can transfer resistance genes to other antimicrobial classes, further complicating the dissemination and global emergence of multi-drug resistant (MDR) *E. coli* (Zhou et al., 2023).

All these factors have influence in the treatment of UTI in community. In this scenario, the Infectious Diseases Society of America (IDSA) recommends the use of fosfomycin trometamol, nitrofurantoin, and pivmecillinam for the treatment of uncomplicated community acquired UTI, while cephalosporins are recommended as alternative therapy. On the other hand, ciprofloxacin and other fluoroquinolones are no longer recommended for the treatment of urinary tract infections due to its side effects and due to the high prevalence of resistance to these antimicrobials. Still, fluoroquinolones continue to be used in the treatment of these infections in Brazil (Gupta et al., 2011; da Silva et al., 2021).

Considering that antibiotic resistance profiles differ between regions, identifying local resistance patterns is crucial for the appropriate management of urinary tract infections (UTIs). Therefore, this study aimed to characterize the MLST, serotype, virulence and resistance genes of resistant UPEC samples isolated from UTI in community.

## Materials and methods

### Samples

*E. coli* strains were isolated from urine samples collected from women attended by Public Health as part of routine examinations in primary care service in a city in Southern Brazil from June 2016 to May 2018. Only isolates resistant to at least one fluoroquinolone and/or producing extended-spectrum *β*-lactamases (ESBLs) were included, as the aim was to investigate high-risk UPEC isolates with clinically significant resistance mechanisms.

### Identification and antimicrobial susceptibility

Bacterial identification and antimicrobial susceptibility testing (AST) were performed using the VITEK® 2 automated system (bioMérieux, USA), employing the GN ID card and AST 238 card. Prior to testing, isolates were subcultured on CHROMID CPS Elite Agar ® and incubated at 35–37 °C for 18–24 h. The antimicrobial susceptibility test included 14 *β*-lactam and non-β-lactam antibiotics: ampicillin, amoxicillin/clavulanate, ceftriaxone, cefepime, ertapenem, meropenem, nalidixic acid, ciprofloxacin, norfloxacin, gentamicin, amikacin nitrofurantoin, trimethoprim-sulfamethoxazole and piperacillin-tazobactam. The interpretation was performed according to the CLSI 2017 (Clinical and Laboratory Standards Institute) criteria. *E. coli* ATCC25922 was used as a quality control strain.

### Analysis of genetic variability

A single colony of each isolate was inoculated in Tryptic Soy Broth overnight and the suspension was then used for DNA extraction by the DNA extraction kit (Invitrogen, Carlsbad, CA). Enterobacterial Repetitive Intergenic Consensus (ERIC-PCR) was performed as previously described by Versalovic et al. (1991). Reactions were prepared in a final volume of 25 μL containing 1 × reaction buffer, 2.5 mM MgCl₂, 200 μM dNTPs, 0.5 μM of each primer, 1 U Taq DNA polymerase, and 50 ng of template DNA. PCR cycling conditions were initial denaturation at 95 °C for 5 min; 35 cycles of denaturation at 94 °C for 1 min, annealing at 52 °C for 1 min, and extension at 72 °C for 2 min; and a final extension at 72 °C for 10 min. Products were resolved on 1.5% agarose gels stained with ethidium bromide and visualized under UV illumination. Banding patterns were analyzed using BioNumerics software (version 4.6), applying the Dice coefficient and UPGMA clustering with 3% position tolerance. Bands below 200 bp or above 12,000 bp were excluded. Isolates with ≥85% similarity were considered genetically related.

### Whole-genome sequencing

Isolates were grown on Mueller-Hinton agar for 18–24 h at 37 °C. A single colony was inoculated into 2 mL Luria–Bertani broth and incubated for 12 h at 37 °C. DNA was extracted using the PureLink™ Genomic DNA Kit according to the manufacturer’s instructions. DNA concentration was measured using the Qubit dsDNA BR Assay Kit (Invitrogen), and purity was assessed by spectrophotometry (A260/A280 ratio of 1.8–2.0). A minimum of 1–10 ng of DNA with a concentration ≥0.5 ng/μL was required for library preparation. Paired-end libraries (150 bp × 150 bp) were prepared using the Illumina Nextera XT DNA Library Prep Kit. Sequencing was performed on the Illumina NextSeq platform (mid-output flow cell). Reads were quality filtered to retain bases with PHRED ≥30, and the sequencing runs consistently yielded >80% of bases above Q30. Average genome coverage ranged from 40 × to 100 × across isolates.

### Bioinformatic analysis

Genome assemblies was performed by the CLC Genomics Workblench version 7.0 (Aarhus, Denmark). Multilocus sequence type (MLST), resistome and virulome were identified using bioinformatics tools MLST 2.0, PlasmidFinder 2.0, ResFinder 3.1 and VirulenceFinder 2.0 available in Center for Genomic Epidemiology.^1^

### Statistical analysis

The chi-square test was performed to evaluate the presence of virulence genes in ESBL-producing isolates or those exhibiting fluoroquinolone resistance mechanisms (plasmid-mediated or mutations). The statistical test was performed using SPSS v.25 software (IBM, USA) with a significance level of 95% (*p* < 0.05).

## Results

**Figure 1:**
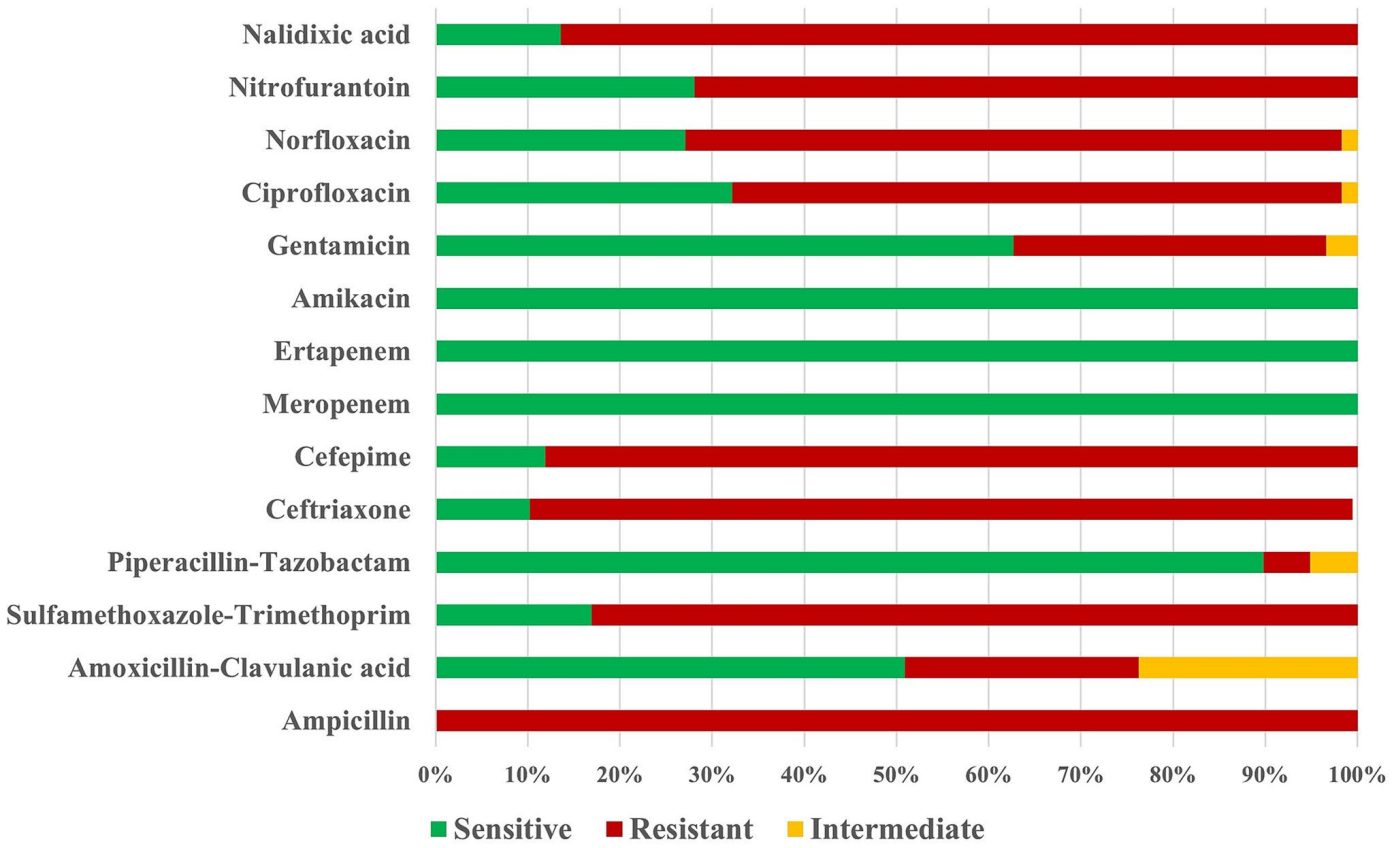
Antimicrobial susceptibility of *E. coli* isolates resistant to at least one fluoroquinolone and/or ESBL producers from urinary tract infections.

In this study, 59 *E. coli* isolates resistant to at least one fluoroquinolone and/or ESBL producers were included for genome analysis. The antimicrobial susceptibility teste showed high resistance rates to ampicillin (100%), nalidixic acid (96.6%), ceftriaxone (83.3%), cefepime (81.5%), ciprofloxacin (81.5%), norfloxacin (81.5%), sulfamethoxazole/trimethoprim (75.9%), and nitrofurantoin (71.9%). On the other hand, the isolates exhibited low resistance rates to gentamicin (33,9%), amoxicillin/clavulanic acid (25.4%), and piperacillin/tazobactam (3.7%). All isolates were sensitive to meropenem, ertapenem, and amikacin (Figure 1).

**Figure 2:**
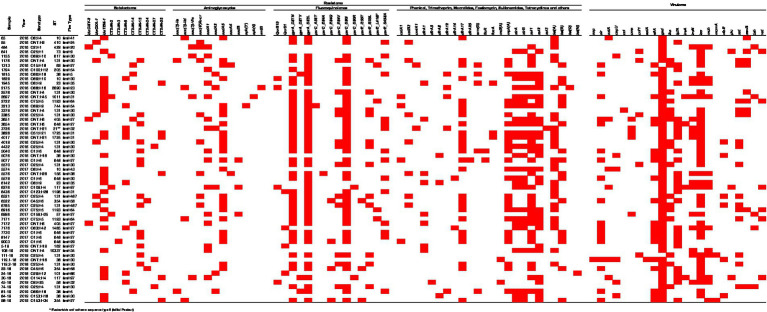
Resistome and virulome of *E. coli* isolates resistant to at least one fluoroquinolone and/or ESBL producers from urinary tract infections.

Detailed results of whole-genome sequencing, resistome and virulome of each isolate are shown in Figure 2 and Supplementary Table 1. The isolates belong to different STs and exhibited a diversity of serotypes. A total of 25 different STs were identified, with most *E. coli* isolates belonging to ST131 (22.2%), ST648 (13.0%), and ST38 (7.4%). Additionally, 29 different serotypes were detected, with the most common being O25: H4 (16.7%) and O1: H6 (11.1%).

The *β*-lactam resistance was mainly mediated by CTX-M (77.8%), TEM-1B (44.4%), OXA-1 (11.1%), and CMY-2 (3.7%). Among ESBL genes, eight variants were identified (CTX-M-2, −3, −8, −14, −15, −24, −27, and −55), and CTX-M-15 was the most frequently isolated in 25.9% of the isolates, followed by CTX-M-8 (14.8%), CTX-M-14 (11.1%), and CTX-M-55 (11.1%). Two isolates (3.7%) exhibited coexistence of the CTX-M-8 and CTX-M-55 genes.

Regarding the presence of QRDR, 98.1% of the isolates have at least one of the *gyrA*, *parC*, and *parE* mutations. Three types of mutations were found in *gyrA*, with the S83L (87.0%) and D87N (75.9%) mutations being the most prevalent among the isolates. In *parC*, six different types of mutations were detected, with S80I being the most frequent (79.6%). Among the four types of mutations found in *parE*, S458A was the most common (25.9%). Observing the presence of PMQR determinants, *qnrB19* was found in four isolates (7.4%), and *qnrS1* in three (5.6%).

Resistance genes associated with other antimicrobial were also identified, being the most frequent: *sul1* (61.1%) and *sul2* (48.1%) which confer resistance to sulfonamides; *aac (3)* (83.1%), *aac(6′)-lb-cr* (86.4%), *aph(3′)*, and *aph (4)* (98.3%) related to aminoglycosides; *tetA* (42.3%) and *tetB* (31.5%) to tetracyclines; *dfrA-17* (48.4%) and *drfA-1* (13.0%) to trimethoprim; genes related to fosfomycin resistance (*fosA*) was found in 5.6% of the isolates.

Several virulence factors were also identified, with 13 of them being frequently associated with urinary tract infections (UTIs). The most common gene was *iss* (72.2%), followed by *IpfA* (48.1%), *eilA* (33.3%), *iha* (31.5%), *sat* (27.7%), and *iroN* (24.1%). Most of the isolates (70.4%) have up to three virulence factor genes, while 25.9% have four to six genes. The virulence genes *iha* and *sat* were significantly more prevalent in isolates carrying *parE* mutations (*p* < 0.001 and *p* = 0.001, respectively). No other virulence genes showed statistically significant differences among CTX-M–producing isolates or those harboring plasmid-mediated quinolone resistance genes (*qnrB19* or *qnrS1*), nor among isolates with *gyrA* and *parC* mutations. Nevertheless, certain virulence genes exhibited a statistical trend toward higher occurrence in isolates presenting these resistance mechanisms (Supplementary Table 2).

Plasmid was present in almost all samples (Supplementary Table 1), except in one. The most frequent plasmid was IncF (95.0%) followed by Col (63.33%) and Incl-1 (25.0%), moreover other types of plasmids (IncX, p0111, IncR, IncQ2 and IncA/C2) were found at lower frequencies.

## Discussion

UTI is one of the main infections affecting community patients and is the second most common bacterial infection treated in primary care, especially in women of childbearing age (Rowe and Juthani-Mehta, 2023; Flores-Mireles et al., 2022). UPEC is responsible for a large percentage of these infections and is often associated with multidrug resistance to antimicrobials. The primary classes of first-line treatment for UTIs include nitrofurantoin quinolones and beta-lactams, therefore, the increased spread of resistance genes to these drugs in the community is becoming a major public health concern (Rowe and Juthani-Mehta, 2023; Mittal et al., 2022).

As expected, resistance rates to aminoglycosides and carbapenems were low, as these classes of antimicrobials are not usually the first choice for community acquired UTI treatment. On the other hand, high resistance percentages were found for penicillins, cephalosporins, quinolones, and sulfonamides. The rapid dissemination of ESBLs is an alarming trend and is considered one of the world’s major health threats. Among these, CTX-M enzymes have emerged as the predominant type of ESBLs encountered in clinical settings. It has been reported that *E. coli* isolates producing CTX-M often carry resistance to multiple classes of antibiotics, including cotrimoxazole, aminoglycosides, and quinolones, further complicating treatment options (Mittal et al., 2022; Meletis and Tzampazidou, 2022).

For *β*-lactams, the high resistance rate to ampicillin can be associated with the *blaTEM-1* gene, which confers resistance to penicillin and first- and second-generation cephalosporins. Moreover, most isolates exhibiting resistance to third- and fourth-generation cephalosporins carried *blaCTX-M* gene. However, a small subset of isolates showed cephalosporin resistance without detectable ESBL genes, possibly due to gene mutations or multi-drug efflux pumps hyperexpression. The global emergence of CTX-M-producing *E. coli* is driven by the rapid dissemination of the gene *blaCTX-M* located on highly mobilizable elements, such as plasmids and transposons. Over 172 variants of CTX-M have been identified, which cluster into five groups: CTX-M-1, −2, −8, −9, and −25 groups. In our study, the *blaCTX-M* genes found in the highest proportions belong to groups 1 (*blaCTX-M-15* and *blaCTX-M-55*), 8 (*blaCTX-M-8*), and 9 (*blaCTX-M-14*) (Meletis and Tzampazidou, 2022; Moghnieh et al., 2023). The CTX-M-1 and CTX-M-9 groups are frequently associated with *E. coli* in various regions of the world, with CTX-M-15 being the predominant variant in the human population globally. CTX-M-8 is less common globally compared to other variants, such as CTX-M-15 and CTX-M-14. However, it has been reported in some UPEC isolates, especially in certain regions of Latin America and other locations where this variant is more prevalent (Lartigue and Leflon-Guibout, 2023; Rocha et al., 2022; Oliveira and Morais, 2021).

ST131, ST648, and ST38 clones are internationally known and are associated with various severe infections, including UTIs. *E. coli* ST131 is the most widely recognized clone due to its association with UTIs and antimicrobial resistance, particularly due to the production of CTX-M-15 (Meletis and Tzampazidou, 2022; Moghnieh et al., 2023). Studies indicate that 70 to 90% of ESBL-producing ST131 strains carry the *blaCTX-M-15* gene, which illustrates its contribution to cephalosporin resistance. Additionally, the O25 serotype is frequently linked to this lineage, contributing to its virulence and capacity for dissemination. The results of this study reinforce this global epidemiology (Oliveira and Morais, 2021; Horcajada et al., 2022).

The co-production of CTX-M-8 and CTX-M-55 is not a common phenomenon and was first described in poultry samples in Brazil in 2018. This may indicate significant selective pressure in different environments due to the frequent use of antibiotics (Jacoby and Strahilevitz, 2021; Soncini et al., 2022). Both isolates belong to ST1725, demonstrating that *E. coli* clones can spread through the population, causing infections and carrying plasmids with different resistance genes. Indeed, the diversity of resistance genes can be observed in the studied strains, as they exhibit high percentages of various genes that confer resistance to sulfonamides, aminoglycosides, tetracyclines, trimethoprim, and fosfomycin. Multidrug resistance is widely discussed in various studies and is associated with the presence of plasmids (Soncini et al., 2022; Fernández-Reyes and Rodríguez-Martínez, 2023; Piotrowski and Kowalski, 2022).

Despite the use of quinolones being discouraged after warnings from the Food and Drug Administration (FDA) about the serious side effects of fluoroquinolone antibiotics (joint or tendon pain, muscle weakness, confusion, hallucinations, and low blood sugar levels), quinolones are the most prescribed antibiotics for uncomplicated urinary tract infections in several countries (Carattoli and Villa, 2022). Resistance to these antimicrobial agents is usually due to mutations in the drug targets. Isolates that were resistant to nalidixic acid, ciprofloxacin, and norfloxacin frequently carried multiple QRDR mutations in *gyrA* (S83L, D87N) and *parC* (S80I, E84V), consistent with high-level fluoroquinolone resistance observed in our study. These resistance mechanisms are frequently detected, especially in MDR international high-risk clones, such as in *E. coli* ST131, ST648, and ST38 (Carattoli and Villa, 2022; Bernasconi and Endimiani, 2022). These features are usually seen in high-risk clones because they can persist and spread for a longer period in the community and are associated with different infections. Additionally, high-risk clones can acquire additional resistance determinants, such as the *qnrB19* and *qnrS1* genes, which, despite conferring low levels of fluoroquinolone resistance, can contribute to the dissemination of resistance in these strains (Choby and Weinstein, 2021). The *aac(6′)-Ib-cr* gene is also known to confer resistance to quinolones and aminoglycosides, and its presence in 13% of the isolates is a significant finding, as it has been increasingly associated with higher resistance to these classes of antibiotics worldwide (Wu et al., 2023; Strahilevitz and Jacoby, 2022). Furthermore, isolates carrying certain aminoglycosides, sulfonamides and other resistance determinants remain phenotypically sensitive, which may reflect gene truncation, low expression, or the presence of silent mutations. These discrepancies highlight the limitations of current databases in capturing all chromosomal and regulatory resistance mechanisms, reinforcing the need for complementary phenotypic assays and curated genomic annotations.

The identification of multiple virulence factors in *E. coli* isolates demonstrates the pathogenic potential of these strains. Numerous studies have shown that the virulence factors *iss*, *eilA*, *iha*, *sat*, and *iroN* are common in UPEC, and their epidemiology varies across different regions of the world. These genes are associated with serum resistance, virulence regulation, adhesion, toxin secretion and iron acquisition, contributing to host invasion and infection (Nakayama and Sato, 2022; Smith and Lewis, 2023). In Europe and United States, the prevalence of *iss* ranges from 25 to 60% in UPEC isolates, while the prevalence of *ipfA* is between 20 and 30% across various regions, including the Americas, Europe and Asia. Therefore, the virulence factors identified in this study are consistent with global patterns (Smith and Lewis, 2023; Hooton and Scholes, 2022; Vann and Sokurenko, 2022). Moreover, the virulence gene profile, where most isolates present up to three genes and a significant subset carries four to six genes, reveals substantial variability in the virulence potential of *E. coli* strains. Moreover, isolates belonging to high-risk clones (ST131, ST648, ST38) exhibited greater overlap between quinolone resistance determinants and virulence genes compared to other STs. This variation may reflect differences in the clinical outcomes of infections caused by these strains, with those harboring more virulence factors potentially leading to more severe or recurrent infections (Vann and Sokurenko, 2022; Ranjbar and Tabrizi, 2022).

Plasmids of several different incompatibility types have been identified in *E. coli*. Our data demonstrates that IncF plasmids are the most common plasmid type in fluoroquinolone resistant and ESBL-producing UPEC, and this is consistent with previous studies. In European countries, the prevalence of IncF plasmids in UPEC may reach 70%. Additionally, other types of plasmids are also common in *E. coli*, such as IncI1, IncN, IncA/C, and IncQ. IncI1 plasmids are common in UPEC isolates in Brazil, with prevalences of 20–30%. Analysis of co-occurrence patterns showed that isolates carrying these plasmids and QRDR mutations, particularly *parE* mutations, tend to harbor a higher number of virulence factors such as*, iha, sat* and other resistance determinants, especially within ST131, supporting their role as vehicles for multidrug resistance and virulence traits (Lalioui and Clermont, 2023; Fernandes and Lincopan, 2021).

In conclusion, this study underscores the growing challenge of multidrug-resistant *E. coli* in UTIs and highlights significant issues related to resistance and adaptability. The extensive spread of resistance genes and virulence factors requires a multifaceted approach, including enhanced surveillance, targeted treatment strategies, and public health interventions.

## Data Availability

The datasets presented in this study can be found in online repositories. The names of the repository/repositories and accession number(s) can be found in the article/Supplementary material.

## Footnotes

1 http://genomicepidemiology.org/

## Supplementary material

SUPPLEMENTARY TABLE 1 Whole genome sequencing, antimicrobial susceptibility and plasmidome of *E. coli* isolates resistant to at least one fluoroquinolone and/or ESBL producers from urinary tract infections.

SUPPLEMENTARY TABLE 2 Statistical analysis of *E. coli* isolates resistant to at least one fluoroquinolone and or ESBL producers from urinary tract infections.
